# Being a young radiation oncologist in Poland: results of a multi-institutional survey

**DOI:** 10.1007/s13187-021-01998-0

**Published:** 2021-03-30

**Authors:** Ewa Pawlowska, Bartłomiej Tomasik, Mateusz Spałek, Artur J. Chyrek, Aleksandra Napieralska

**Affiliations:** 1 Department of Oncology and Radiotherapy, Faculty of Medicine, Medical University of Gdansk, Gdansk, Poland; 2 Department of Biostatistics and Translational Medicine, Medical University of Lodz, Lodz, Poland; 3 Department of Radiation Oncology, Medical University of Lodz, Lodz, Poland; 4 Postgraduate School of Molecular Medicine, Medical University of Warsaw, Warsaw, Poland; 5 Department of Soft Tissue/Bone Sarcoma and Melanoma, Maria Sklodowska-Curie National Research Institute of Oncology, Warsaw, Poland; 6 Brachytherapy Department, Greater Poland Cancer Center, Poznan, Poland; 7 Maria Sklodowska-Curie National Research Institute of Oncology Gliwice Branch, Gliwice, Poland

**Keywords:** Radiation oncology, Young specialists, Education, Employment, Survey

## Abstract

In 2018, Polish Society of Radiation Oncology formed a young section (yPTRO), dedicated to radiation oncologists under the age of 40. To evaluate their current situation, an anonymous, nationwide, online survey was carried out. Thirty-two-item-based questionnaire investigated young radiation oncologists’ perception of employment, workload, education, malpractice lawsuits, scientific research, and board exam. A total of 44 physicians responded to the questionnaire, yielding a response rate of 25%. Results of the survey identified the main problematic areas. In general, young radiation oncologists in Poland are overloaded with bureaucracy. They complain on spending too much time at work and lack work-life balance. The risk of being sued for medical error is threatening two-thirds of responders in everyday work. Compensation is not satisfying for nearly half of the survey participants. Nearly all young radiation oncologists continue education and participate in national and international educational events. Forty-eight percent of responders do scientific research alongside clinical work. However, the perception of young radiation oncologists on the board exam is alarming and requires further discussion. Fifty-five percent of the survey participants think that current form of the exam is not appropriate. Hopefully, 75% of physicians feel fairly evaluated. The presented report is the first of its kind in Poland. Issues mentioned in our questionnaire will help newly formed yPTRO to develop strategic priorities for the upcoming years.

**Supplementary Information:**

The online version contains supplementary material available at 10.1007/s13187-021-01998-0.

## Introduction

Radiation oncology (RO) is a rarely chosen medical specialty with a relatively low number of specialists worldwide. It remains unknown for both general public and other medical professionals. Thus, the leading international RO societies, namely the European Society for Radiotherapy and Oncology (ESTRO) and the American Society for Radiation Oncology (ASTRO), play an essential role in creating a sense of community and supporting members from all over the world. Societies provide standards of education and practice in radiotherapy, organize courses and meetings, and facilitate research in RO [1]. In 2018, ESTRO published the results of an online survey for RO professionals under 40 years on national education systems. They received 463 questionnaires. Unfortunately, only few professionals from Poland responded [2]. Taking into consideration that in 2018 there were 592 radiation oncologists in Poland and according to the data in the Polish national physicians’ registry, 176 of them were 40-and-younger, such underrepresentation is worrisome [3, 4].

The same year, Polish Society of Radiation Oncology (PTRO) formed a young section (yPTRO) for RO specialists and trainees under the age of 40. Such sections are popular among National Societies, e.g., AIRO Giovani (Italy), SYROG (Spain), SFjRO (France), or yDEGRO (Germany) [5–8]. They address the special needs of young physicians for employment, education, and research. Moreover, young sections monitor feedback on standards for training and board certification.

The first and only national survey on RO training in Poland was performed in 2007 [9]. In 2018, yPTRO decided to conduct two surveys to evaluate the situation of young radiation oncologists in Poland. The first one was dedicated to RO trainees (results were published in 2020 by Napieralska et al. [10]).

Hence, we present the results of the second survey on the present situation of young RO specialists (yROS) in Poland.

## Methods

### Survey design

An anonymous survey was designed by the yPTRO. Six volunteers (radiation oncology trainees and young specialists from five radiation oncology departments) prepared a sampling plan, evaluated and revised questions through a series of remote discussions. The format was consequently modified according to suggestions until it reached unanimous approval. The unvalidated questionnaire was self-designed using Google Forms (available at https://docs.google.com/forms/). It comprised of 30-single-choice questions, each with a box for comments, one multiple-choice question, and one rating question (the translated version of the survey is attached as a supplementary material). Percentages were calculated using returned questionnaires. Results were divided into six sections: (1) employment and salary, (2) workload, (3) education, (4) malpractice lawsuits, (5) scientific research, and (6) board exam. Participants were not required to answer all questions.

### Survey distribution

The survey was launched on 17th October 2018. Invitations were sent via email and across social media (Facebook® platform) to yROS. To increase the number of responses, individual invitations, and reminders, as well as emails to RO departments, were sent over the data collection period, which closed on 9th November 2018. RO trainees were excluded.

### Statistical analysis

The survey results were mostly descriptive. The comparative statistics were limited because of the relatively small sample size. Calculations were done using Microsoft Excel®.

## Results

A summary of answers to all single-choice-questions is available in Supplementary Materials - Table S1

### Participants

A total of 44 yROS responded to the survey, yielding a response rate of 25%. Twenty-five (57%) yROS provided the name of an employer reporting 14 cancer centers (Table 1). All but one declared working in the public healthcare system. In 2018, there were 47 radiotherapy departments in Poland [3]. There were no data on the number of yROS working in each department.

**Table 1:** List of oncological centers represented by the survey participants

Name of the oncological center	Voivodeship	Number of respondents
Greater Poland Cancer Center	Greater Poland	8
Amethyst Radiotherapy Center in Cracow	Lesser Poland	1
Maria Sklodowska-Curie Memorial Cancer Center and Institute of Oncology Cracow Branch	Lesser Poland	1
N. Copernicus Memorial Hospital, Lodz, Poland	Lodz province	1
Lower Silesian Oncology Center Legnica Branch	Lower Silesian	1
Center of Oncology of the Lublin Region St. Jana z Dukli	Lublin province	1
Maria Sklodowska-Curie Memorial Cancer Center and Institute of Oncology Warsaw	Masovian	2
Bialystok Oncology Centre	Podlaskie	1
Gdynia Oncology Centre of the Polish Red Cross Maritime Hospital in Gdynia	Pomeranian	1
University Clinical Centre in Gdansk	Pomeranian	1
Beskid Oncology Center	Silesian	1
Maria Sklodowska-Curie Memorial Cancer Center and Institute of Oncology Gliwice Branch	Silesian	3
Subcarpathian Oncology Center	Subcarpathian	2
Holy Cross Cancer Center	Holy Cross province	1
Not indicated		19
Total		44

### Employment and salary

In Poland physicians who work in hospitals have two main types of work arrangements, namely regular employment or self-employment with an independent contract with a medical unit.

Twenty-three (52%) yROS worked as employees and 18 (41%) were independent contractors. The others (7%) had both types of work arrangements. Thirty-five (80%) yROS declared being satisfied with the current type of employment; however, only 24 (55%) responders could freely choose it. Satisfaction rate was lower among self-contractors (78%) than employees (87%) and those with both types of employment (100%). Eight (18%) yROS, who were not satisfied with the type of employment, complained about the salary (employees) and lack of social benefits (independent contractors).

Forty-one (93%) yROS did not experience problems with finding a job after board certification. Nevertheless, the majority of yROS (*n*=37, 84%) continued work at the department providing their RO training.

Forty-two (95%) yROS responded to the question about general job satisfaction. The vast majority (*n*=32, 76%) were satisfied with the working conditions. None of the contractors complained compared to over 35% unsatisfied employees and 1 unsatisfied person with both types of employment. Negative comments mentioned a necessity to choose work-life balance and a limited number of radiotherapy departments in their neighborhood.

Twenty-three (52%) yROS worked at two or more places, mainly due to financial reasons (*n*=13, 57%). Personal interests were the second reason for having more than one job (*n*=7, 30%).

Thirty-five (80%) yROS worked as radiation oncologists in the gap between finishing training and taking board exams. Only eight physicians (18%) did not have this opportunity.

Twenty-six (59%) yROS were satisfied with their salaries.

### Workload

Most of yROS (*n*=24, 55%) spent at work approximately 40 h per week. Fifteen (34%) yROS declared longer working time, around 50 h a week. The others declared working part-time or up to 30 h per week. Twenty-four (55%) yROS had night shifts at their departments.

**Fig. 1:**
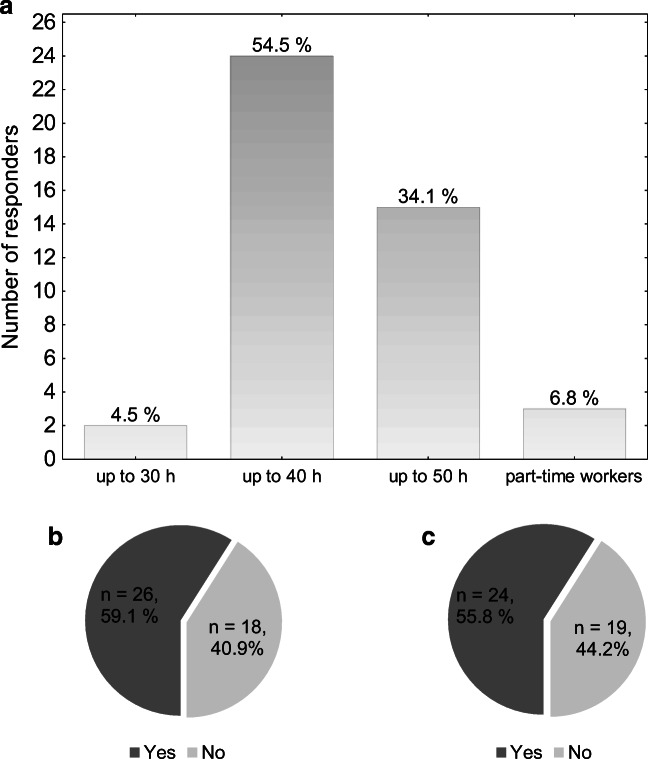
Workload. **a** Average number of working hours per week. **b** In your opinion, do you spend too much time at work? **c** Does job negatively influence your life?

Despite that declared weekly working hours were not alarming and corresponded to the typical 8-h working day, and 26 responders (59%) wrote that, in their opinion, they spend too much time at work. Twenty-four (56%) yROS noticed the negative impact of work on their private lives (Fig. 1).

To assess yROS’ workload, we asked about the number of new admissions per week. Twenty-seven (61%) yROS consulted five to ten new patients per week. Thirteen (30%) yROS declared admitting less than five patients per week, while the others (9%) consulted 11 to 15 new patients per week. More than half of yROS (*n*=26, 59%) thought that their workload is optimal. Ten (23%) responders stated that the number of new patients is too high. Only six (14%) yROS complained about having too few patients. Half of the yROS (*n*=22, 51%) declared having no impact on the number of weekly admissions. In turn, 21 (49%) yROS could decide on the number of new patients per week.

According to the survey results, yROS were overloaded with administrative tasks. Most of the yROS (*n*=21, 48%) spent 51–75% of daily time on these responsibilities. Two yROS declared that paperwork consumes 76–100% of their working hours. Fourteen (32%) yROS declared that administrative responsibilities take from 26 to 50% of daily time. Seven (16%) yROS spent on it less than a quarter of their working time. It means that more than 50% of the survey participants spent more than half a day on nonclinical tasks. Such a workload with paperwork was frustrating for 35 (88%) yROS. Only four (10%) yROS claimed that the number of administrative tasks is reasonable.

### Education

**Fig. 2:**
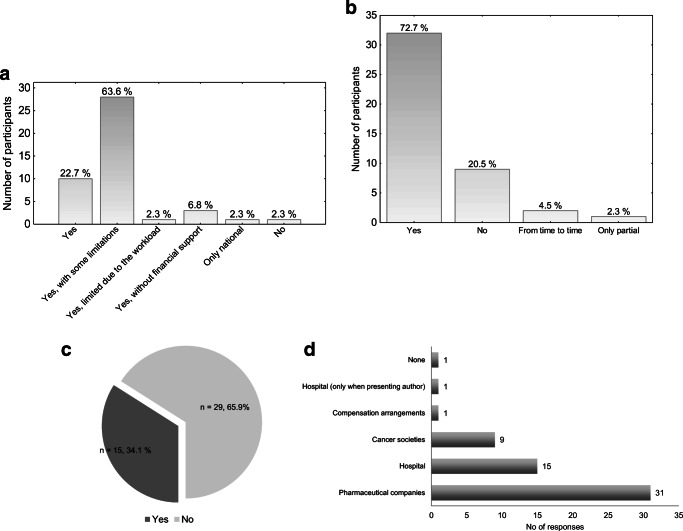
Education. **a** Can you attend national/international educational events? **b** Do you get financial support for participation in educational events? **c** Do you think that the number of educational events you attend is optimal? **d** Sources of reimbursement

Forty-three (98%) yROS participated in national and/or international educational events; however, most of them (*n*=33, 75%) experienced difficulties with accessing such events (Fig. 2). Fifteen (34%) yROS considered the number of attended courses as satisfactory.

Thirty-five (80%) yROS got financial support for educational events, mainly from pharmaceutical companies (*n*=31, 74%), their hospital (*n*=15, 36%), and/or cancer societies (*n*=9, 21%).

The length of educational leave per year was enough in the opinion of 13 (30%) and insufficient for 17 (39%) yROS. Thirteen (30%) yROS working as independent contractors declared no educational leave privilege.

Twenty-six (59%) yROS felt confident with the acquired level of clinical knowledge. All survey participants declared having the possibility to seek for advice/consult within their department.

### Malpractice lawsuits

Thirty (68%) yROS claimed that the risk of being sued for medical errors is on their mind in everyday work. Fourteen (32%) yROS declared that the threat of malpractice lawsuits does not affect their work.

### Scientific research

Nearly half (*n*=21, 48%) of yROS declared doing scientific research alongside with clinical work. For 20 (49%) yROS, the clinical work overload negatively impacted their research.

### Board exam

Twenty-four (55%) yROS thought that the form of the board exam was not reliable, in contrary to 20 (45%) who were satisfied with it. However, most of yROS (*n*=33, 75%) claimed that they were fairly evaluated on their exam. Nine yROS (20%) considered the board exam as not valid.

**Fig. 3:**
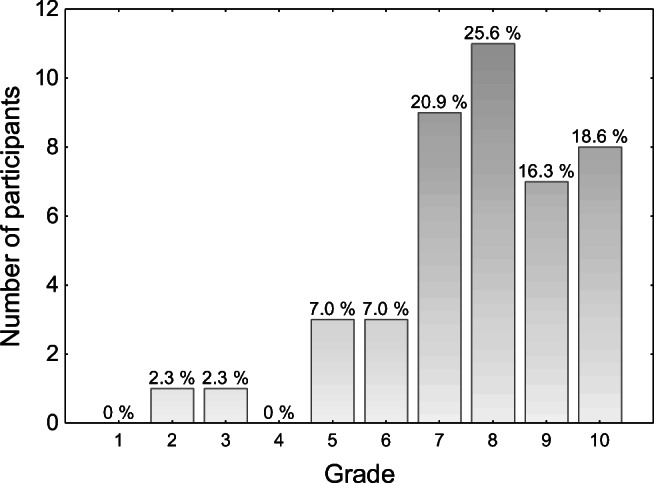
Difficulty of the board exam (1 = very easy and 10 = very tough)

The board exam was considered to be tough. Participants were asked to assess its difficulty from 1 (very easy) to 10 (very tough). Median value from 43 responses was 8 (interquartile range: 7–10). The answers are presented in Fig. 3.

## Discussion

To the best of our knowledge, this is the first nationwide survey providing a comprehensive overview of the situation of Polish yROS. Previously, in 2007, the survey conducted among RO specialists addressed a different group of participants and was reported only as a short letter [9]. Issues mentioned in our questionnaire will help newly formed yPTRO to develop strategic priorities for the upcoming years.

Results of our study identify some problem areas. On the one hand, most yROS are satisfied with the place of work and form of employment. Moreover, working hours declared by respondents corresponded to the typical 8-h working day. Polish yROS spend less hours weekly at work than their German colleagues. They also less often participate in night shifts [8]. On the other hand, the proportion of yROS complaining about lack of work-life balance is striking, achieving more than 50% of participants. Identification of underlying causes needs more in-depth investigation. yROS are overloaded with bureaucracy that causes frustration in 88% of them. Such imbalance between administrative tasks and clinical duties may cause burnout syndrome. However, data about its prevalence among yROS are scarce. Ciammella et al. conducted an online survey among Italian yROS and identified factors significantly influencing the risk of developing the syndrome such as working position, number of years of practice, work hours per week, lack of cooperation in the team, lack of opportunities for personal development, and uncertainties on the working perspectives [11]. Up to now, there are no data on the frequency of burnout syndrome among Polish RO specialists and further research on this topic is warranted. Nevertheless, many initiatives can be implemented as preventive strategies, some of them at the local level (such as task restructuring, work evaluation and supervision, and management support) [12]. As yPTRO, we plan to organize courses enhancing job competencies, improving coping skills, and managing negative emotions. Moreover, as a national society, we plan to monitor by repeatable surveys the prevalence of the burnout syndrome among our members.

Paperwork overload may be related to the fear of malpractice lawsuits raised by the two-third of yROS. In Poland, the employment of medical secretaries is still infrequent. Thus, the whole paperwork must be done by a physician. It results in sacrificing expert skills, knowledge, and time that could be used for patient care. However, it is a nationwide problem that requires general legislative reforms to be implemented by the government. Hopefully, there are some initiatives helping physicians to deal with the risk of malpractice lawsuits. Regional medical councils in each voivodeship organize medical law courses and offer free of charge legal assistance for its members. Moreover, RO trainees during residency are obliged to take part in a dedicated medical law course.

Another issue for further discussion is salary. Again, there are no data on salaries of Polish radiation oncologists, but 41% of yROS were dissatisfied with work compensation in our survey. This is in line with the results of an ASTRO study in which satisfaction with salary among American RO specialists reached mean score 6.3 (scale 1–10, 1 = very dissatisfied and 10 = very satisfied) [13]. Consequently, the low salary was the most common reason for having a second job. Problem with unsatisfactory work compensation is rather complex, and solving it is beyond yPTRO; however, Best et al. identified business and financial management as one of the perceived gaps in the transition to practice in radiation oncology. As a solution, they suggest formal and informal teaching, mentorship, and educational resources [14]. Up to now, there are no courses dedicated yROS on to the topic of financial management. We plan to fill this gap by preparing webinars or online courses accessible to all yROS.

Another important area of discussion is education and personal development. RO is a rapidly developing specialty, and keeping up to date with knowledge is essential. Every year, ESTRO and ASTRO organize various educational events, courses, and congresses. Moreover, there are dozens of international and national oncological meetings. Almost all yROS (98%) declared taking part in educational events. However, for the three-fourth of them, participation in such activities was limited by extra conditions. This may explain why 66% of yROS are not satisfied with the number of courses they could attend. Hopefully, most of yROS get financial support for postgraduate education that might be crucial in the case of insufficient salary. Another obstacle in self-development may be the limited access to the educational leave, considered as scarce by 39% of yROS. All this, taken together, gives us a possible reason for uncertainty of 36% yROS about possessing the knowledge sufficient for work. As yPTRO, we observe a lack of courses and educational events dedicated to yROS. To fill this gap, since 2018, we are working on a mentoring program with short, focused on prespecified problem fellowships in polish departments, allowing for self-development, gaining experience, accelerating implementation of new RO techniques across the country and enhancing a sense of community. Moreover, we are preparing to start an educational platform with free online courses and webinars on statistics or clinical trials in radiation oncology. One of our goals is to implement a program of financial support for young specialists not only for participation in educational events but also for research.

Board exam was not the main topic of our survey and mentioned in only three questions. However, results are not encouraging and should be discussed with PTRO and Polish National Consultant in RO. Despite the fact that 75% of survey participants believe to be fairly evaluated on their board exam, more than half of physicians think that its current form is not appropriate.

This study’s limitations include a low response rate (25%) that might introduce ascertainment bias. It reduces the representativeness of the sample and the possibility of generalizing the survey results. One of the reasons of such a low response rate may be the lack of a database of Polish yROS. Due to the General Data Protection Regulation, it is not possible to acquire such data from physician registries. Interestingly, the yPTRO survey on RO trainees achieved a response rate of 68% [10]. Comparison to other national surveys among yROS is difficult as most of them enrolled both trainees and young specialists [8, 15]. According to the literature, online surveys tend to achieve even lower response rates than received 25% [16]. The reason of low responsiveness of yROS could be explained by the workload and lack of time [17]. Such unwillingness of Polish yROS to participate in surveys was present in the largest European survey conducted by ESTRO [2]. This problem requires particular attention and actions taken by both yPTRO and PTRO. What can be done from our perspective is creating an updated database of yROS containing all important information, e.g., department, age, and mailing address allowing for direct contact for future studies and surveys.

Another weakness could be the lack of survey validation; however, such validated tools do not exist.

## Conclusions

yROS in Poland face many problems in their everyday work and need support in keeping a work-life balance. Further, nationwide questionnaires on burnout syndrome and salaries are warranted. yPTRO will represent both RO trainees and yROS in discussions on identified issues.

## Supplementary Information

ESM 1 (DOCX 27 kb)

ESM 2 (DOCX 32 kb)

## Data Availability

Data and material are available upon Reviewer or Editor request.
