# Supplementary material for: Being a young radiation oncologist in Poland: results of a multi-institutional survey
Source: J Cancer Educ. 2021 Mar 30;37(6):1614–20. doi: 10.1007/s13187-021-01998-0 (PMC9681692; doi:10.1007/s13187-021-01998-0)
Supplement: Supplementary file 1 — (DOCX 27 kb) [file 13187_2021_1998_MOESM1_ESM.docx]

Name of radiotherapy (RT) department:

1. Type of work arrangement:
   1. Regular employment
   2. Independent contractor
   3. Other (…)
2. Are you satisfied with the type of your work arrangement?
   1. Yes
   2. No
   3. Other (…)
3. Did you have freedom in selecting a type of work arrangement?
   1. Yes
   2. No
   3. Other (…)
4. Did you have problems with finding a job after a board exam?
   1. Yes
   2. No
   3. Other (…)
5. Do you continue to work at the department providing your RT training?
   1. Yes
   2. No
   3. Other (…)
6. Are you satisfied with your place of work?
   1. Yes
   2. No
   3. Other (…)
7. What is your average number of working hours per week?
   1. Up to 30
   2. Up to 40
   3. Up to 50
   4. Part-time worker
8. Do you feel like spending too much time at work?
   1. Yes
   2. No
   3. Other (…)
9. Do you think that work has a negative impact on your private life?
   1. Yes
   2. No
   3. Other (…)
10. Do you have night shifts in your primary workplace?
    1. Yes
    2. No
    3. Other (…)
11. Do you have a second job?
    1. Yes
    2. No
    3. Other (…)
12. If you have a second job please provide a reason for it:
    1. Financial
    2. Personal interests
    3. Continuing previous employment
    4. Other (…)
13. Are you satisfied with a salary at your primary workplace?
    1. Yes
    2. No
    3. Other (…)
14. What is the average number of your new admissions per week (RT planning):
    1. <5
    2. 5-10
    3. 11-15
    4. 16-20
    5. >20
15. In your opinion, the number of new admissions is:
    1. Too low
    2. Adequate
    3. Too high
    4. Other (…)
16. Can you decide on the number of new admissions?
    1. Yes, that is my own decision
    2. Yes, I can ask for it
    3. No
    4. Other (…)
17. Can you participate in national and international educational events?
    1. Yes
    2. Yes, with some limitations
    3. No
    4. I am not interested
    5. Other (…)
18. Do you consider the number of educational events you participate as sufficient?
    1. Yes
    2. No
    3. Other (…)
19. Do you get financial support for participation in educational events?
    1. Yes
    2. No
    3. Other (…)
20. If you get financial support for participation in educational events, it comes from (choose all that apply):
    1. Pharmaceutical companies
    2. Hospital/RT department
    3. Cancer societies
    4. Other (…)
21. Do you think that the number of educational leave days is sufficient?
    1. Yes
    2. No
    3. Not applicable (independent contractor)
    4. Other (…)
22. Do you feel confident about the level of clinical knowledge you possess?
    1. Yes
    2. No
    3. Other (…)
23. Do you have the opportunity to consult problematic patients within your department?
    1. Yes
    2. Yes, with some limitations
    3. No
    4. Other (…)
24. What proportion of daily working time do you spend on paperwork?
    1. 0-25%
    2. 26-50%
    3. 51-75%
    4. 76-100%
25. The load of paperwork:
    1. Irritates me
    2. Do not bother me
    3. Other (…)
26. Are you afraid of being sued for medical error?
    1. Yes
    2. No
    3. Other (…)
27. Do you do scientific research alongside with clinical work?
    1. Yes
    2. No
    3. Other (…)
28. Do you think that clinical work overload negatively impacts your research?
    1. Yes
    2. No
    3. Other (…)
29. Do you think that the current form of the board exam is appropriate?
    1. Yes
    2. No
    3. Other (…)
30. Did you feel fairly evaluated on the board exam?
    1. Yes
    2. No
    3. Other (…)
31. Rate the difficulty of the board exam (1=very easy, 10=very tough)
32. Did you have the opportunity to work as a radiation oncologist in the gap between finishing training and taking the board exam?
    1. Yes
    2. No
    3. Other (…)
