# Supplementary material for: Being a young radiation oncologist in Poland: results of a multi-institutional survey
Source: J Cancer Educ. 2021 Mar 30;37(6):1614–20. doi: 10.1007/s13187-021-01998-0 (PMC9681692; doi:10.1007/s13187-021-01998-0)
Supplement: Supplementary file 2 — (DOCX 32 kb) [file 13187_2021_1998_MOESM2_ESM.docx]

| **QUESTION** | **ANSWERS** | **NUMBER OF RESPONSES (%)** |
| --- | --- | --- |
| Type of work arrangement: | Regular employment | 23 (52%) |
|  | Independent contractor | 18 (41%) |
|  | Both | 3 (7%) |
| Are you satisfied with the type of your work arrangement? | Yes | 35 (80%) |
|  | No | 6 (14%) |
|  | Lack of stabilization and social benefits | 1 (2%) |
|  | Low salary | 1 (2%) |
|  | Just changing my arrangement | 1 (2%) |
| Did you have freedom in selecting a type of work arrangement? | Yes | 24 (55%) |
|  | No | 20 (45%) |
| Did you have problems with finding a job after a board exam? | Yes | 41 (93%) |
|  | No | 3 (7%) |
| Do you continue to work at the department providing your RT training? | Yes | 37 (84%) |
|  | No | 7 (16%) |
| Are you satisfied with your place of work? | Yes | 32 (76%) |
|  | No | 7 (17%) |
|  | From time to time | 1 (2%) |
|  | I chose balance between private life and work | 1 (2%) |
|  | There are no other departments nearby | 1 (2%) |
| What is your average number of working hours per week? | Up to 30 | 2 (5%) |
|  | Up to 40 | 24 (55%) |
|  | Up to 50 | 15 (34%) |
|  | Part-time worker | 3 (7%) |
| Do you feel like spending too much time at work? | Yes | 26 (59%) |
|  | No | 18 (41%) |
| Do you think that work has a negative impact on your private life? | Yes | 24 (56%) |
|  | No | 19 (44%) |
| Do you have night shifts in your primary workplace? | Yes | 24 (55%) |
|  | No | 20 (45%) |
| Do you have a second job? | Yes | 23 (52%) |
|  | No | 21 (48%) |
| If you have a second job please provide a reason for it: | Financial | 13 (57%) |
|  | Personal interests | 7 (30%) |
|  | Continuing previous employment | 3 (13%) |
| Are you satisfied with a salary at your primary workplace? | Yes | 26 (59%) |
|  | No | 18 (41%) |
| What is the average number of your new admissions per week (RT planning): | <5 | 13 (30%) |
|  | 5-10 | 27 (61%) |
|  | 11-15 | 4 (9%) |
| In your opinion, the number of new admissions is: | Too high | 10 (23%) |
|  | Adequate | 26 (59%) |
|  | Too low | 6 (14%) |
|  | Hard to say | 1 (2%) |
|  | Too high in some months | 1 (2%) |
| Can you decide on the number of new admissions? | No | 22 (51%) |
|  | Yes | 21 (49%) |
| Can you participate in national and international educational events? | Yes | 10 (23%) |
|  | Yes, with some limitations | 28 (64%) |
|  | Yes, limited due to the workload | 1 (2%) |
|  | Yes, without financial support | 3 (7%) |
|  | Only national | 1 (2%) |
|  | No | 1 (2%) |
| Do you consider the number of educational events you participate as sufficient? | Yes | 15 (34%) |
|  | No | 29 (66%) |
| Do you get financial support for participation in educational events? | Yes | 32 (73%) |
|  | No | 9 (21%) |
|  | From time to time | 1 (2%) |
|  | Only partial | 2 (5%) |
| Do you think that the number of educational leave days is sufficient? | Yes | 13 (30%) |
|  | No | 17 (39%) |
|  | Not applicable (independent contractor) | 13 (30%) |
|  | Did not know about it | 1 (2%) |
| Do you feel confident about the level of clinical knowledge you possess? | Yes | 26 (59%) |
|  | No | 16 (36%) |
|  | I learn everyday | 2 (5%) |
| Do you have the opportunity to consult problematic patients within your department? | Yes | 33 (75%) |
|  | Yes, with some limitations | 11 (25%) |
| What proportion of daily working time do you spend on paperwork? | 0-25% | 7 (16%) |
|  | 26-50% | 14 (32%) |
|  | 51-75% | 21 (48%) |
|  | 76-100% | 2 (5%) |
| The load of paperwork: | Irritates me | 35 (88%) |
|  | Do not bother me | 3 (8%) |
|  | Is sometimes annoying | 1 (2%) |
|  | Is reasonable due to the law regulations | 1 (2%) |
| Are you afraid of being sued for medical error? | Yes | 30 (68%) |
|  | No | 14 (32%) |
| Do you do scientific research alongside with clinical work? | Yes | 21 (48%) |
|  | No | 23 (52%) |
| Do you think that clinical work overload negatively impacts your research? | Yes | 20 (49%) |
|  | No | 19 (46%) |
|  | Not applicable | 2 (5%) |
| Do you think that the current form of the board exam is appropriate? | Yes | 20 (45%) |
|  | No | 24 (55%) |
| Did you feel fairly evaluated on the board exam? | Yes | 33 (75%) |
|  | No | 9 (20%) |
|  | Hard to say | 2 (5%) |
| Did you have the opportunity to work as a radiation oncologist in the gap between finishing training and taking the board exam? | Yes | 30 (70%) |
|  | No | 7 (16%) |
|  | Yes, continuing my work arrangement as an employee | 5 (12%) |
|  | No, I worked instead in Clinical Oncology Department | 1 (2%) |

**Tab. S1** Summary of responses to single-choice-questions (percentages may not add up to 100 due to rounding)
